# Heat Priming and Heat Stress Enhance Transgenerational Heat Tolerance in the Early Growth Stages of *Oryza sativa* L. Progeny

**DOI:** 10.3390/plants14111593

**Published:** 2025-05-23

**Authors:** Younghwan Ju, Juyoung Choi, Sungho Yun, Jun Ichi Sakagami

**Affiliations:** 1 The United Graduate School of Agricultural Sciences, Kagoshima University, Korimoto 1-21-24, Kagoshima 890-0065, Japan; joh7598@naver.com (Y.J.); jychoi8519@gmail.com (J.C.); 2 Digitalomics Research Center, Korea Basic Science Institute (KSBI) Ochang Center, 161, Yeongudanji-ro, Ochang-eup, Cheongwon-gu, Cheongju-si 28119, Chungcheongbuk-do, Republic of Korea; sungho@kbsi.re.kr; 3 Faculty of Agriculture, Kagoshima University, Kagoshima 890-0065, Japan

**Keywords:** heat priming, transgenerational heat tolerance, oxidative stress, biomass accumulation

## Abstract

Rice (*Oryza sativa* L.) is a staple crop that provides essential nutrients and energy; however, it is sensitive to heat stress, posing a threat to sustainable productivity. Heat stress can cause delayed germination in progeny, increased oxidative stress, reduced biomass accumulation, and excessive water loss. Notably, heat stress memory induced through heat priming can be inherited, potentially strengthening heat tolerance in subsequent generations. This study examined the effects of heat priming and heat stress on delayed germination, shoot length, and shoot fresh and dry weight under elevated-temperature conditions. The results showed that while heat stress delayed germination in progeny, heat priming significantly accelerated germination rates. Furthermore, heat stress elevated oxidative stress levels, thereby hindering biomass synthesis. In contrast, heat priming helped maintain low levels of reactive oxygen species (ROS) and malondialdehyde (MDA), contributing to greater biomass accumulation. These findings suggest that heat priming enhances transgenerational heat tolerance in rice, leading to faster germination, higher biomass accumulation, and improved ROS homeostasis in progeny.

## 1. Introduction

Rice (*Oryza sativa* L.) is a major staple crop, feeding more than half of the global population and supplying essential nutrients, including antioxidants, vitamins, carbohydrates, proteins, and fats [1]. However, rising global temperatures [2] present significant threats to rice productivity [3,4,5]. For instance, a 1 °C rise in global mean temperature is projected to reduce rice yields by approximately 3.2% [6].

Germination, the transition from a heterotrophic seed to an autotrophic seedling, is a critical phase for seedling growth and organ development [7,8]. Germination is regulated by energy metabolism using storage compounds, such as starch, which is a major source of energy for germination by glycolysis, the pentose phosphate pathway, and the tricarboxylic acid cycle (TCA) [8]. Starch synthesis can be limited by temperature stress, which may lead to a decline in grain quality [9]. Also, heat stress during this stage can lead to excessive water loss, oxidative damage, stunted shoot and root growth, and even seedling mortality. Additionally, the number of viable seedlings may decline under heat stress, potentially reducing the final rice yield [10]. Therefore, understanding the physiological responses of rice to heat stress is imperative for ensuring stable crop production.

Under heat stress conditions, impaired electron transport chains in chloroplasts and mitochondria result in the excessive generation of reactive oxygen species (ROS) due to disrupted cellular redox homeostasis. This ROS overproduction can cause oxidative stress, resulting in lipid peroxidation, protein denaturation, and DNA damage. To mitigate this, plants activate antioxidant defense pathways that include enzymes such as ascorbate peroxidase (APX), catalase (CAT), and superoxide dismutase (SOD). While ROS play essential roles in stress signaling and tolerance, their excessive accumulation is detrimental, emphasizing the importance of maintaining ROS homeostasis for heat stress tolerance [11,12,13].

Plants have the ability to “remember” previous stress events, thereby enabling more rapid and effective responses to subsequent stresses. This phenomenon, known as priming, alters the plant’s response to future stress exposures [14,15,16]. Heat priming contributes to improved tolerance by limiting excessive ROS accumulation [11,12,13]. Previous studies have shown that stress priming involves histone methylation, modifications in regulatory proteins, and other epigenetic mechanisms, and that the resulting heat tolerance can be inherited by the next generation [14,15,16,17].

In this study, we hypothesized that heat priming induces transgenerational heat tolerance, thereby enhancing rice germination and early seedling growth under heat stress conditions. To test this hypothesis, we examined the germination rate, shoot length, biomass accumulation, ROS production, and lipid peroxidation in the progeny of two rice varieties, N22 and IR64, that were heat-primed during the flowering stage. These traits were selected as they represent key physiological indicators of early-stage heat stress response and seedling vigor. Our aim was to evaluate whether priming-induced stress memory could be inherited and functionally expressed in the next generation. By integrating physiological and biochemical measurements, this study provides a mechanistic understanding of how parental exposure to heat stress may enhance stress tolerance in progeny. Our findings provide insights into the epigenetic effects of heat priming on transgenerational heat tolerance and offer promising avenues for mitigating heat stress, particularly in the context of global climate change.

## 2. Results

### 2.1. Experimental Design Overview

**Figure 1:**
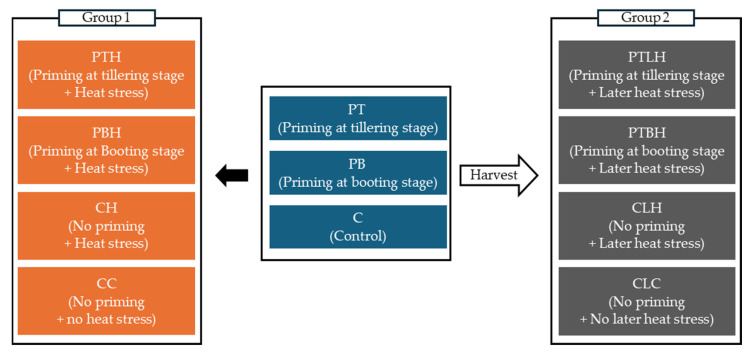
Schematic diagram of seed preparation and treatments. The blue boxes represent the heat-priming treatments applied before any heat stress. Heat priming was applied for five days either at the tillering stage (PT) or the booting stage (PB), while the control group (C) received no priming. After priming, rice plants were divided into two major groups. Group 1 was subjected to heat stress before harvest to examine the effects of heat priming on the same generation. This group included four treatments: PTH (tillering-stage priming with heat stress), PBH (booting-stage priming with heat stress), CH (no priming with heat stress), and CC (no priming without heat stress). Group 2 was maintained without heat stress after priming and used to investigate the heat stress response in the progeny. This group consisted of four treatments: PTLH (progeny from tillering-stage heat priming for five days, exposed to later heat stress for three days), PBLH (progeny from booting-stage heat priming for five days, exposed to later heat stress for three days), CLH (progeny from non-primed parents, exposed to later heat stress for three days), and CLC (progeny from non-primed parents, not exposed to heat stress). Solid black arrows indicate heat stress applied within the same generation, and white-center hollow arrows indicate postharvest progression to the next generation for stress evaluation.

To investigate the transgenerational effects of heat priming under different developmental stages, we established four experimental progeny groups derived from plants subjected to different heat priming and stress conditions (Figure 1).

### 2.2. Changes in Germination Parameters

Table 1 presents germination parameters at 28 °C, including germination percentage (GP), time for 50% germination (T50), and mean germination time (MGT), for progenies of heat-primed N22 and IR64 with heat stress during flowering. GP was recorded every 12 h, with the first germination observed 36 h after imbibition. In both varieties, germination of the CH (no heat priming with heat stress at flowering for seven days) treatment began at 48 h after imbibition (hai), whereas germination in heat-primed treatments and the CC (no heat priming with no heat stress) group started earlier, at 36 hai. Compared to CC, CH showed the greatest GP reduction at 48 hai in N22 and 60 hai in IR64. In N22, PTH (heat priming at the tillering stage for five days with heat stress at flowering for seven days) had a higher GP compared to PBH (heat priming at the booting stage for five days with heat stress at flowering for seven days) at 48 hai, whereas in IR64, PBH had a higher GP relative to PTH at 60 hai. T50 and MGT were significantly higher in CH than in CC for both varieties, but they decreased in PTH and PBH compared to CH. In N22, T50, and MGT were lower in PTH than in PBH, whereas in IR64, PBH had lower values compared with PTH. Heat priming significantly influenced GP, T50, and MGT across all time points, as confirmed via a three-way ANOVA. At 60 hai, a significant interaction between variety and treatment was observed, indicating differential responses to heat priming.

### 2.3. Biomass Parameters

**Figure 2:**
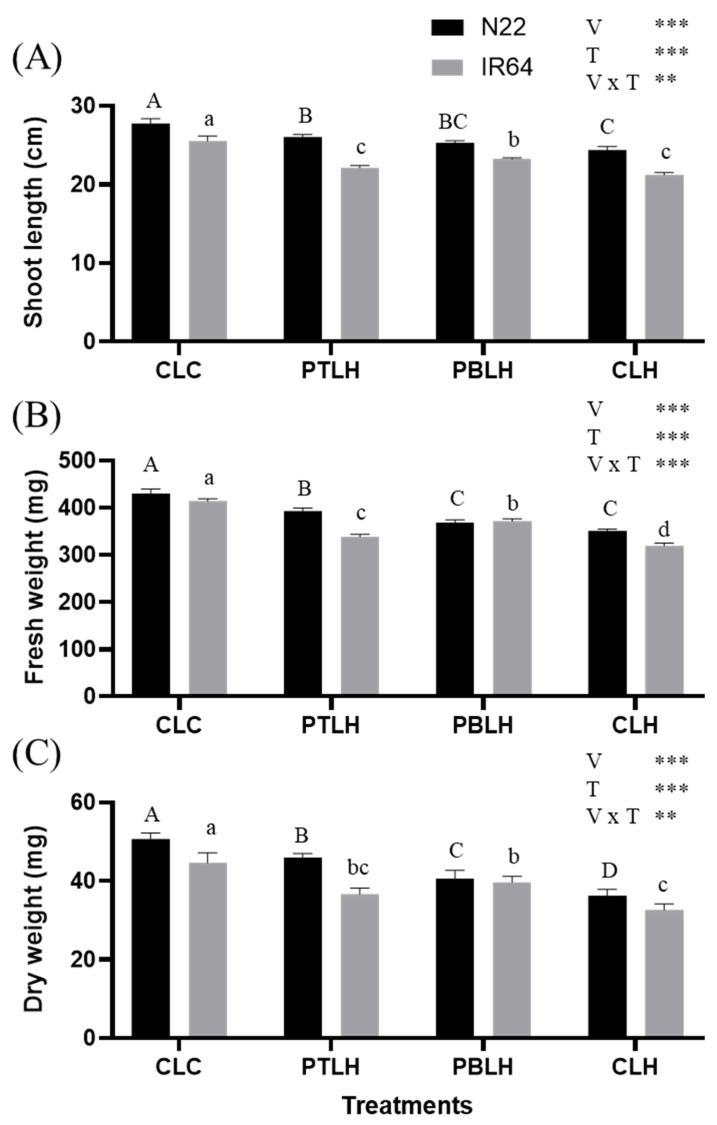
Biomass parameters, including shoot length (**A**), shoot fresh weight (**B**), and shoot dry weight (**C**), of the progenies of heat-primed rice (cv. N22 and IR64) under heat stress. Heat stress was applied at the 14-day seedling stage with 16 h of light (38 °C) and 8 h of darkness (28 °C) for three days. Asterisks, ** *p* < 0.01, and *** *p* < 0.001) indicate significant interaction effects between variety (V) and treatment (T), as determined by two-way ANOVA. Tukey’s HSD test (*p* < 0.05) was performed to compare treatment means within each variety. Identical uppercase letters (A, B, C, D) indicate no significant difference among treatments in N22, and identical lowercase letters (a, b, c, d) indicate no significant difference among treatments in IR64. Abbreviations: CLC: progeny from non-primed parents, not exposed to heat stress; PTLH: progeny from tillering-stage heat priming for five days, exposed to later heat stress for three days; PBLH: progeny from booting-stage heat priming for five days, exposed to later heat stress for three days; CLH: progeny from non-primed parents, exposed to later heat stress for three days.

Biomass parameters, including shoot length (SL), fresh weight (FW), and dry weight (DW), were measured after three days of heat stress (Figure 2). In both varieties, SL, FW, and DW decreased significantly under heat stress compared to CLC (progeny from non-primed parents, exposed to later heat stress for three days), but the negative effects were significantly alleviated by transgenerational heat priming. The most pronounced alleviation was observed in N22 under the PTLH treatment (progeny from tillering-stage heat priming for five days, exposed to later heat stress for three days) across all three parameters. In contrast, IR64 showed the greatest mitigation under PBLH (progeny from booting-stage heat priming for five days, exposed to later heat stress for three days). According to two-way ANOVA revealed that SL, FW, and DW were strongly influenced by variety, treatment, and their interaction.

### 2.4. H_2_O_2_ and MDA Content

**Figure 3:**
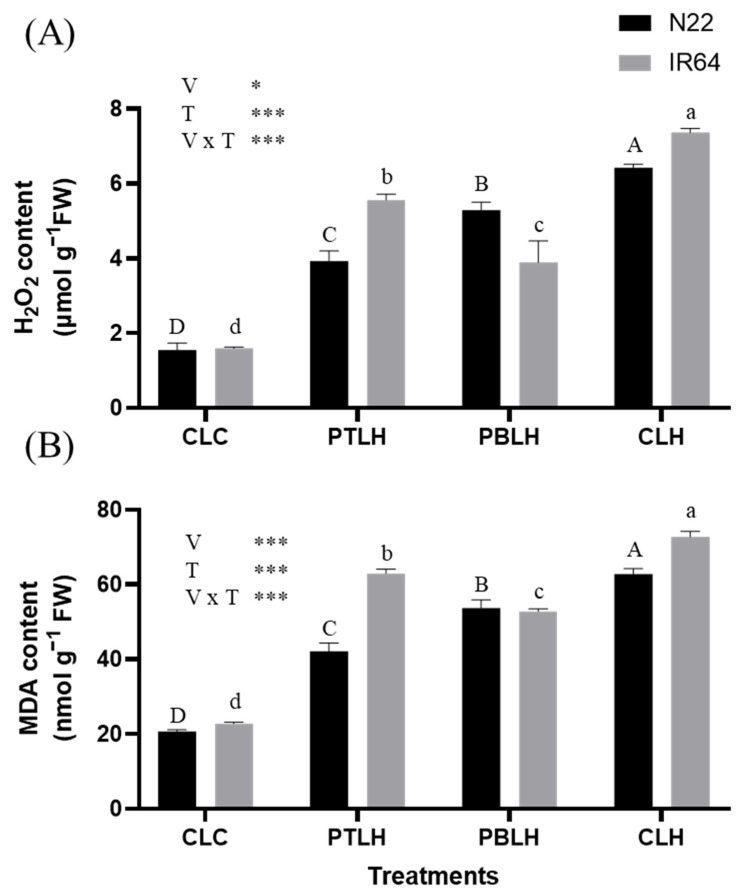
Hydrogen peroxide (H_2_O_2_) (**A**) and malondialdehyde (MDA) (**B**) contents in the progenies of heat-primed rice (cv. N22 and IR64) under heat stress. Heat stress was applied at the 14-day seedling stage with 16 h of light (38 °C) and 8 h of darkness (28 °C) for three days. Asterisks (* *p* < 0.05 and *** *p* < 0.001) indicate significant interaction effects between variety (V) and treatment (T), as determined by two-way ANOVA. Tukey’s HSD test (*p* < 0.05) was performed to compare treatment means within each variety. Identical uppercase letters (A, B, C, D) indicate no significant difference among treatments in N22, and identical lowercase letters (a, b, c, d) indicate no significant difference among treatments in IR64. Abbreviations: CLC: progeny from non-primed parents, not exposed to heat stress; PTLH: progeny from tillering-stage heat priming for five days, exposed to later heat stress for three days; PBLH: progeny from booting-stage heat priming for five days, exposed to later heat stress for three days; CLH: progeny from non-primed parents, exposed to later heat stress for three days.

ROS were quantified by measuring hydrogen peroxide (H_2_O_2_) content, and lipid peroxidation was measured according to malondialdehyde (MDA) content (Figure 3). Three days of heat stress significantly elevated the H_2_O_2_ and MDA content in both varieties across all treatments compared to CLC. In the CLH (progeny from non-primed parents, exposed to later heat stress for three days) treatment, H_2_O_2_ levels increased by 314% in N22 and 361% in IR64. In contrast, the PTLH treatment resulted in smaller increases of 153% and 248% in N22 and IR64, respectively, while the PBLH treatment led to increases of 241% in N22 and 144% in IR64. Similarly, the MDA content in CLH increased by 203% and 219% in N22 and IR64, respectively, compared with CLC. However, in PTLH, the MDA levels increased by only 103% and 176% in N22 and IR64, respectively, and in PBLH, the increases were limited to 159% and 131% in N22 and IR64, respectively.

## 3. Discussion

To assess heat priming’s impact on mitigating heat stress effects on progeny germination, parameters such as GP, T50, and MGT were measured (Table 1). Transgenerational heat stress delayed germination at 48 and 60 hai in N22 and IR64, respectively. Notably, all germination parameters were influenced by treatment, indicating that both rice varieties experienced transgenerational effects from heat priming and heat stress.

During germination, marking the initial stage in a plant’s life cycle, essential processes occur, including mRNA translation and DNA repair, and germination is followed by seedling growth and organ development [7,8,18]. Heat stress during grain filling can lead to spikelet sterility and delayed germination, without affecting progeny seed vigor [19,20]. In the present study, T50 and MGT increased in both varieties, although no significant differences were observed among the treatments, supporting the notion that heat stress delays germination without reducing seed vigor.

Epigenetic modifications, such as histone modifications, DNA methylation, chromatin remodeling, and RNA production, are involved in germination [21,22]. These modifications may mitigate heat stress effects on progeny germination by regulating the expression of genes involved in hormonal signaling, dormancy release, and ROS homeostasis, all of which play critical roles in seed germination under stress conditions, suggesting an association with the observed germination parameters (Table 1). While this interpretation remains hypothetical, further investigation into the epigenetic regulation of transgenerational germination responses would be both valuable and scientifically intriguing.

Abiotic stresses, including heat, drought, and salt, markedly impact crop productivity [23]. Heat stress can irreversibly damage growth, photosynthesis, respiration, water relations, and membrane stability. It may also trigger ROS hyperaccumulation, leading to oxidative damage, cell death, growth inhibition, and potential seedling death [10,24,25]. Although antioxidant enzymes, such as superoxide dismutase, catalase, and glutathione reductase, typically regulate ROS, heat stress can damage these scavengers, resulting in ROS accumulation [26]. Low ROS concentrations can serve as secondary messengers, whereas high concentrations cause oxidative stress. Among ROS, H_2_O_2_ is notably stable and diffusive, with higher cell membrane permeability [25]. Excessive ROS levels also elevate MDA content, causing protein and nucleic acid damage. Thus, low ROS and MDA levels under heat stress indicate heat tolerance [27,28]. Heat priming induces numerous heat stress responses, including changes in histone protein, heat shock protein, and heat shock factor expression. Studies have shown that heat tolerance acquired through priming can be inherited, enabling the progeny of heat-primed rice plants to exhibit enhanced tolerance [12,16,29].

Under heat stress conditions, heat-primed progeny displayed reduced SL, FW, and DW compared to CLC (Figure 2). Notably, the SL, FW, and DW of heat-primed N22 and IR64 rice plants were higher compared to CLH, highlighting the heat-stress-mitigating effect of priming. This suggests that heat-primed progeny can produce more biomass under heat stress. Heat stress elevated the H_2_O_2_ and MDA levels (Figure 3), but these levels were reduced in PTLH and PBLH compared to CLH, reflecting heat priming’s alleviative effect. ROS accumulation from heat stress can lead to lipid peroxidation [30,31]; therefore, the relative increases in SL, FW, DW, H_2_O_2_, and MDA in PTLH and PBLH compared to CLH likely indicate increased heat tolerance due to transgenerational priming effects (Figure 2 and Figure 3).

Although heat stress reduced biomass and increased oxidative damage parameters, the responses varied between the rice varieties, as confirmed via a two-way ANOVA. In CLH, the H_2_O_2_ and MDA increases were smaller in N22 than in IR64. Two-way ANOVA also revealed the differing mitigating effects of heat priming between the cultivars: in N22, PBLH had a stronger alleviative effect compared to PTLH, whereas in IR64, PTLH’s effects surpassed those of PBLH. These observations may reflect the innate differences in heat tolerance between N22 and IR64, which show tolerance and susceptibility, respectively. Additionally, the high responsiveness of N22 during the tillering stage may represent an avoidance strategy to cope with early-stage stress, while the strong response of IR64 during the reproductive stage could be associated with its greater vulnerability and stress memory formation at that phase. These observations suggest that the optimal timing of priming treatments may need to be tailored based on cultivar-specific characteristics.

The results of the present study demonstrated that heat tolerance acquired through heat priming in rice can be inherited, helping maintain heat stress memory across generations. Heat priming effectively reduced the delay in germination observed in the progeny of heat-stressed rice. Additionally, heat-primed rice maintained lower ROS and MDA levels under heat stress, contributing to increased SL, fresh weight, and DW. These results highlight the transgenerational relief of oxidative stress achieved through heat priming, which enables rice plants to accumulate more biomass. Although our study demonstrates the inheritance of heat priming effects in the immediate progeny, it remains unclear whether these effects persist beyond a single generation. Several studies in model and crop species have reported partial transmission of stress-induced epigenetic changes to subsequent generations (e.g., F2), though these effects are often attenuated or reversible [15,32,33]. Further studies are required to determine the stability and functional significance of such transgenerational memory in rice.

## 4. Materials and Methods

### 4.1. Seed Preparation

Seeds of *Oryza sativa* L. varieties N22 and IR64 were surface-sterilized using 1% (*v*/*v*) sodium hypochlorite (NaClO) for 20 min and then rinsed thoroughly ten times with distilled water until NaClO was completely removed. The sterilized seeds were transferred to sterile glass Petri dishes (90 mm diameter) containing 25 seeds (*n* = 10) and wet filter paper as a moisture source and incubated in the dark at 25 °C for 24 h in a growth chamber (INCUBATOR SIB-35, SANSYO, Hamamatsu, Japan) to induce germination. Uniformly germinated seeds were then transplanted into nursery trays and maintained in a glasshouse located outdoors. During this 1-week nursery period, temperature, humidity, and light were not artificially controlled and followed the natural environmental cycle. Seedlings were watered daily to ensure adequate soil moisture. After one week, seedlings with uniform growth were selected and transplanted into plastic pots (256 × 234 × 297 mm) filled with commercial bed soil (Ryoto Fertilizer Co., Ltd., Oita, Japan) containing NKP (0.6:1.5:1.0) per pot. The experiment was conducted at an outdoor laboratory of Kagoshima University (31°34′23.5″ N, 130°32′35.0″ E). At 45 days after transplanting, an additional 10 g of NPK (1:1:1) fertilizer was applied to each pot. Each pot contained five rice seedlings, and five replicate pots were used per treatment in a completely randomized block design (CRBD).

Heat priming was conducted for five days in a greenhouse where the average temperature was approximately 2–3 °C higher than the ambient temperature. Heat-priming treatment was applied at either at the tillering stage (PT) or booting stage (PB). After priming, the plants were divided into two major experimental groups (Figure 3). The first group was used to study the effect of heat priming and heat stress on progeny seed germination. These plants were subjected to 7 days of heat stress at the flowering stage under conditions that were 4–5 °C higher on average than the ambient temperature by transferring them back to the glasshouse. After stress application, the plants were returned to the outdoor environment for seed maturation. The second group was maintained under outdoor conditions until harvest without exposure to additional heat stress. All harvested seeds were dehydrated in a shaded greenhouse at 30–35 °C and 40–60% relative humidity for two weeks and then stored in sealed bags at 4 °C in the dark prior to use in progeny experiments. The first group consisted of four treatments based on heat priming and heat stress conditions: PTH: heat priming at the tillering stage for five days with heat stress at flowering for seven days, PBH: heat priming at the booting stage for five days with heat stress at flowering for seven days, CC: no heat priming with no heat stress. Temperature and humidity were continuously monitored during the entire experiment using remote sensors (Long-Range Wireless Connection Logger, Telemoni, Tokyo, AXEL, Japan) (Table S1).

### 4.2. Germination Parameters

Seeds from the first group were sterilized with 1% NaClO for 20 min and then washed. Subsequently, 15 sterilized seeds (*n* = 3) were germinated for 72 h on wet filter paper in darkness at 28 °C (Yamato Scientific Co., Tokyo, Japan), with germination measured every 12 h thereafter. Germination percentage (GP), germination speed (T50), and mean germination time (MGT) were calculated as follows:

GP=Germinated seedsTotal number of seeds 

$$T50=ti+\left[\frac{\left(\frac{n+1}{2-ni}\right)}{\left(nj-ni\right)}\right]\times \left(tj-ti\right),$$

where *N* is the final germinated seed count, and ni and nj are cumulative germinated seed counts at times when *ni* < *N*/2 < *nj* [33].

MGT=∑(n×t)/∑N,

where *n* is the number of seeds germinated each time, *t* is the number of times from the beginning of the experiment, and *N* is the total number of seeds germinated at the termination of the experiment [34].

### 4.3. Later Heat Stress Application

Seeds from the second group (Group 2), which were derived from plants that had undergone different priming treatments had not been subjected to flowering-stage heat stress, were sterilized and germinated as previously described. After germination, uniformly germinated seedlings were transplanted into plastic containers (50 mm wide × 200 mm long × 50 mm deep), with 10 seedlings per container. Three biological replicates were used per treatment. The seedlings were grown in a controlled growth chamber under a 16 h light/8 h dark photoperiod, with light intensity maintained at 500 μmol m^−2^ s^−1^. Day and night temperatures were set at 32 °C and 28 °C, respectively.

After 14 days of growth under these conditions, the seedlings were transferred to a plant growth chamber (LPH-241-S, Biotron NK System, Nippon Medical and Chemical Instruments Co., Ltd., Osaka, Japan) for heat stress treatment. Heat stress was applied by exposing the seedlings to 38 °C during the 16 h light period and 28 °C during the 8 h dark period for three consecutive days.

The purpose of this experiment was to evaluate transgenerational heat stress tolerance in the progeny of heat-primed and non-primed parental plants. Accordingly, seedlings were divided into the following four treatment groups (Figure 1):

(1) PTLH: progeny from tillering-stage heat priming for five days, exposed to later heat stress for three days.
(2) PBLH: progeny from booting-stage heat priming for five days, exposed to later heat stress for three days.
(3) CLH: progeny from non-primed parents, exposed to later heat stress for three days.
(4) CLC: progeny from non-primed parents, not exposed to heat stress.

All containers were placed in the chamber in a completely randomized manner to eliminate positional effects. After the heat stress treatment, the plants were used for further analysis of morphological and physiological responses.

### 4.4. Shoot Length and Shoot Biomass

Shoot length (SL) was measured in centimeters using a ruler and shoot fresh weight (FW) was recorded using an electronic scale (HR-100AZ, A&D Company, Tokyo, Japan) before samples were dried at 70 °C for 72 h to determine shoot dry weight (DW) [35]. All measurements were conducted with three biological replicates.

### 4.5. H_2_O_2_ and MDA Analyses

To measure the ROS, H_2_O_2_, 100 mg of leaves was immediately frozen in liquid nitrogen and then homogenized with 5 mL of 0.1% trichloroacetic acid (TCA; *w*/*v*) using a mortar and pestle. The samples were then centrifuged at 12,000× *g* for 15 min (GR-15R, BECKMAN COULTER, Indianapolis, IN, USA). The supernatant was mixed with 0.5 mL of 10 mM potassium phosphate (pH 7) and 1 mL of 1 M potassium iodide, and absorbance was measured immediately at 390 nm using a Spectronic 200 UV-Vis spectrophotometer using an H_2_O_2_ standard curve [36,37].

MDA was measured as a marker of lipid peroxidation using the thiobarbituric acid (TBA) method [38,39]. Leaves (100 mg) were ground with 1 mL of 0.5% (*w*/*v*) TBA containing 20% (*w*/*v*) TCA using a pre-chilled mortar and pestle. The mixture was then incubated at 95 °C for 30 min. To stop the reaction, the mixture was placed in an ice bath, followed by centrifugation at 10,000× *g* for 10 min. Absorbance was measured at 532 and 600 nm, with MDA equivalents calculated as follows:

MDA equivalentsnmol g−1=[(A532−A600)155,000 ]106

All biochemical measurements were performed with three biological replicates. H_2_O_2_ and MDA were extracted immediately after the three-day heat stress treatment, which was applied after 14 days of seedling growth.

### 4.6. Statistical Analysis

Using RStudio (version 4.2.1), Tukey’s honestly substantial difference test was conducted to separate means, with statistical significance set at *p* < 0.05. Three-way ANOVA was performed using the obtained data. Graphical representations were generated via GraphPad Prism (version 10.3.1).

## 5. Conclusions

In the present study, we demonstrated that heat stress during the reproductive stage in rice induces transgenerational effects that delay germination and reduce seedling vigor, as evidenced by increased ROS and MDA levels and reduced biomass in the progeny. In contrast, heat priming at the tillering and booting stages significantly alleviated these negative effects, resulting in improved germination, reduced oxidative damage, and increased metabolite levels in the progeny. These beneficial effects may be attributed to (1) better membrane integrity, (2) enhanced synthesis of protective metabolites, and (3) more effective antioxidant activity. Our findings highlight the potential of heat priming as a strategy to induce transgenerational stress tolerance, especially in susceptible cultivars such as IR64, and suggest that the timing of priming may need to be optimized depending on genotype. This study also offers practical insights for breeding programs aimed at enhancing heat tolerance in rice. However, the mechanisms underlying these effects remain speculative, as the current study did not directly assess epigenetic modifications. In addition, further research across multiple generations and growth stages, as well as yield-related evaluations under field conditions, will be essential to fully validate the transgenerational benefits of heat priming.

## Figures and Tables

**Table 1:** Germination parameters at 28 °C including germination percentage, time for 50% germination (T50), and mean germination time (MGT) of progenies of heat-primed N22 and IR64 at the tillering or booting stages for five days with heat stress applied for seven days during the flowering stage. Germination percentage was measured every 12 h using 15 seeds with three replicates per group.

Variety	Treatment	Germination Percentage (%)				T50	MGT
		36 hai	48 hai	60 hai	72 hai		
N22	CC	6.67 a	73.33 a	84.44 a	91.11 a	43.7 b	50.3 b
	PTH	8.89 a	46.67 ab	75.56 ab	88.89 a	48.7 ab	54.1 ab
	PBH	11.11 a	28.89 ab	60.00 b	84.44 a	53.0 ab	57.7 ab
	CH	0.00 a	26.67 b	42.22 c	77.78 a	54.6 a	61.3 a
IR64	CC	11.11 a	55.56 a	91.11 a	91.11 a	46.4 b	51.2 b
	PTH	4.44 a	42.22 ab	66.67 b	82.22 a	48.6a b	55.3 a
	PBH	8.89 a	44.44 ab	80.00 ab	82.22 a	46.8 b	52.5 b
	CH	0.00 a	31.11 b	62.22 b	71.11 a	50.8 a	56.3 a
Variety		ns	ns	*	ns	ns	ns
Treatment		*	**	***	*	**	**
Variety × Treatment		ns	ns	*	ns	ns	ns

* *p* < 0.05, ** *p* < 0.01, and *** *p* < 0.001 indicate the interactions between variety and stress calculated using two-way ANOVA. The letter ns indicates no interaction. Tukey’s Honest Significant Difference (HSD) test was conducted to divide treatments of each variety at the level of *p* < 0.05. The same lowercase letter indicates no significant difference. Abbreviation; CC: no heat priming with no heat stress, PTH: heat priming at the tillering stage for five days with heat stress at flowering for seven days, PBH: heat priming at the booting stage for five days with heat stress at flowering for seven days, CH: no heat priming with heat stress at flowering for seven days, hai: hours after imbibition, and MGT: mean germination time.

## Data Availability

The raw data supporting the conclusions of this article will be made available by the authors on request.

## Supplementary Materials

The following supporting information can be downloaded at: https://www.mdpi.com/article/10.3390/plants14111593/s1, Table S1: Temperature during heat priming and heat stress.

## Abbreviations

The following abbreviations are used in this manuscript:

PT	Heat priming at the tillering stage
PB	Heat priming at the booting stage
CC	No heat priming with no heat stress
PTH	Heat priming at the tillering stage with heat stress at the flowering stage for seven days
PBH	Heat priming at the booting stage with heat stress at the flowering stage for seven days
CH	No heat priming with heat stress at the flowering stage for seven days
CLC	Progeny from non-primed parents, not exposed to heat stress
CLH	Progeny from non-primed parents, exposed to later heat stress for three days
PTLH	Progeny from tillering-stage heat priming for five days, exposed to later heat stress for three days
PBLH	Progeny from booting-stage heat priming for five days, exposed to later heat stress for three days
