# Supplementary material for: Heat Priming and Heat Stress Enhance Transgenerational Heat Tolerance in the Early Growth Stages of Oryza sativa L. Progeny
Source: Plants (Basel). 2025 May 23;14(11):1593. doi: 10.3390/plants14111593 (PMC12157927; doi:10.3390/plants14111593)
Supplement: Supplementary file 1 [file plants-14-01593-s001.zip › plants-3629569-supplementary.pdf]

Supplementary Table S1 Temperature during heat priming and heat stress

Cultivar	Temperature level	Control	PTH	Control	PBH	Control	HS
IR64	Maximum	30.2	34.7	31.4	34.5	33.0	48.0
	Minimum	21.3	21.3	22.6	23.2	25.8	26.5
	Average	27.2	29.5	26.4	29.2	28.5	33.5
N22	Maximum	30.2	34.7	32.4	35.5	33.2	48.7
	Minimum	21.3	21.3	24.1	25.7	26.1	26.4
	Average	27.2	29.5	26.9	29.2	29.3	33.2

All values are shown in °C. Abbreviations: PTH: heat priming at the tillering stage with heat stress; PBH: heat priming at the booting stage with heat stress applied during the flowering stage; HS: heat stress.
